# Hidden biodiversity of Amazonian white-sand ecosystems: two distinctive new species of *Utricularia* (Lentibulariaceae) from Pará, Brazil

**DOI:** 10.3897/phytokeys.169.57626

**Published:** 2020-12-04

**Authors:** Paulo Minatel Gonella, Rafael Gomes Barbosa-Silva, Andreas S. Fleischmann, Daniela C. Zappi, Paulo Cesar Baleeiro, Caroline Oliveira Andrino

**Affiliations:** 1 Universidade Federal de São João del-Rei, Departamento de Ciências Exatas e Biológicas, Campus Sete Lagoas, Rodovia MG-424, km 47, Sete Lagoas, MG, 35701-970, Brazil; 2 Instituto Nacional da Mata Atlântica, Av. José Ruschi, 4, Santa Teresa, ES, 29500-000, Brazil; 3 Instituto Tecnológico Vale, Rua Boaventura da Silva 955, Nazaré, 66055-090, Belém, PA, Brazil; 4 Museu Paraense Emílio Goeldi, Coord. Botânica, Av. Perimetral 1901, 66077-830, Belém, PA, Brazil; 5 Botanische Staatssammlung München, Menzinger Strasse 67, D-80638, Munich, Germany; 6 Programa de Pós-Graduação em Botânica, Instituto de Ciências Biológicas, Universidade de Brasília, DF, 70910-900, Brazil; 7 University of Queensland, Brisbane, QLD, 4072, Australia

**Keywords:** Amazon savannas, *campinaranas*, carnivorous plants, deforestation, taxonomy, *Utricularia ariramba*, *Utricularia jaramacaru*, wetlands

## Abstract

As deforestation and fire move forward over pristine vegetation in the Amazon, many species remain undiscovered and may be threatened with extinction before being described. Here, we describe two new species of *Utricularia* (Lentibulariaceae) collected during recent fieldwork in an area of white-sand vegetation in the eastern Amazon Basin named Campos do Ariramba. Further herbarium revision revealed that both species were first collected over 60 years ago in the same area, remaining unnamed until now. The new species, named *U. ariramba***sp. nov.** and *U. jaramacaru***sp. nov.**, are placed in U. sect. Aranella and U. sect. Setiscapella, respectively. We provide full descriptions, illustrations, photographs, a distribution map, and taxonomic discussion for both species. Additionally, we provide a preliminary list of Lentibulariaceae from the Campos do Ariramba. Both new species are assessed as Vulnerable, however, yet known only from a few collections each, highlighting the urgency and importance of fieldwork and taxonomic revisions in the Amazon biogeographic region in order to provide essential data for the conservation of both known and still unknown biodiversity.

## Introduction

Brazil is an extremely diverse country, home to the greatest floristic diversity in the world, in addition to being one of the best documented tropical countries in terms of its flora (Forzza et al. 2012; Flora do Brasil 2020 under construction). However, Brazil leads the number of new plant species described yearly (RBG Kew 2016; Cheek et al. 2020), showing that its vast territory still needs to be explored and studied if we are to attain a better understanding of the true dimension of its biodiversity.

Large remote areas of Brazil, especially those difficult to access, still lack taxonomic surveys and are in their majority concentrated in the Amazon Rainforest biome (Oliveira et al. 2016; BFG 2018). Regarded as the most biodiverse rainforest in the world, this region has fewer scientific collections in relation to other Brazilian biomes, with a strong bias of collection effort around large urban centers (Nelson et al. 1990) and along navigable rivers, while over 40% of its total area remains under-sampled and poorly studied (Schulman et al. 2007). Knowledge is even scarcer if one considers the herbaceous plants that grow in open Amazonian vegetation, as the majority of inventories still focus on woody plants (Miranda 1993; Miranda et al. 2002, 2006; Devecchi et al. 2020). Much faster than we are able to provide suitable studies regarding the Amazonian biodiversity, the rapid increase of the deforestation reaching these unexplored areas is potentially causing the extinction of a considerable proportion of undescribed plant species (Stroop et al. 2020).

Although the Amazon is predominantly known for its exuberant evergreen lowland rainforest, there are patches of open areas of outstanding biological diversity, such as the Pantepui (highland vegetation), the Amazonian *canga* (ferruginous *campo rupestre*), inselbergs, *campinaranas* (white sand vegetation) and Amazonian *savannas* (Pires and Prance 1985; Gröger 1994; Prance 1996; BFG 2015; Adeney et al. 2016; Barbosa-Silva et al. 2016, 2020; Mota et al. 2018; Costa et al. 2019; Zappi et al. 2019; Andrino et al. 2020; Devecchi et al. 2020; Fonseca-da-Silva et al. 2020), altogether representing approximately 5% of the Amazon biome in Brazil (IBGE 2020).

All of the above-mentioned open vegetation areas in the Amazon have oligotrophic, acidic soils (consisting of bare sandstone, ferruginous or granite escarpments, or alluvial plains of white sands) with the presence of seasonally or perennially wet to flooded areas (García-Villacorta et al. 2016). In contrast to the surrounding Amazon lowland forests, these habitats have a scattered vegetation cover, often herbaceous or at the most shrubby, and comprise very exposed sites with lower vegetation cover and competition, representing “islands” within the Amazon forest (Prance 1996). These conditions, especially low availability of nitrogen and phosphorus, favor the occurrence of carnivorous plants (Givnish et al. 2018), and indeed several species of *Drosera* L. (Droseraceae), *Genlisea* A.St.-Hil. and *Utricularia* L. (Lentibulariaceae) can be found in those areas (Rivadavia et al. 2009; Fleischmann 2012a; Fleischmann et al. 2017).

The genus *Utricularia* is the most diverse of three genera of the carnivorous plant family Lentibulariaceae (Lamiales, Eudicots), with over 240 species currently accepted, presenting centers of diversity in the Neotropics and northern Australia, where most of its species are associated with seasonally wet areas of savanna vegetation (Taylor 1989; Fleischmann 2012b, 2015; Jobson et al. 2018). In Brazil, *Utricularia* is represented to date by 67 species (18 endemics), being most diverse in the Cerrado (Central Brazilian Savanna) and the Amazon Rainforest, both with 45 species each (Flora do Brasil 2020 under construction).

*Utricularia* is composed of small to medium-sized herbs, usually associated with wetlands, that can be recognized by the atypical morphology, lacking true roots, presence of leaf-like shoots (phylloclades), and bladder-like structures of foliar origin, the utricles, that inspired its generic epithet. The inflorescences are bracteose racemes, the flowers have a bilobate calyx (except in the early-branching U. sect. Polypompholyx (Lehm.) P.Taylor, and a few members of other lineages, such as *U. flaccida* A.DC. from U. sect. Setiscapella (Barnhart) P.Taylor, which can have a tetramerous calyx), a bilabiate personate corolla (snapdragon flower-type), with a spur, two stamens and ovary with central-free placentation (Taylor 1989; Jobson et al. 2003). *Utricularia* shows a great diversity of habitat types and life forms, occurring as aquatics (affixed or free-floating), terrestrials, lithophytes, rheophytes, and epiphytes (Taylor 1989).

Taylor (1989) presented the most comprehensive revision of the genus to date, classifying the accepted species into subgenera and sections. About 30–40 species (depending on species concepts) have been described after Taylor’s monograph (for compiling works, see Fleischmann 2012b, 2015; Jobson et al. 2018), and the infrageneric classification has undergone a few changes based on molecular phylogenetic data (Jobson et al. 2003; Müller and Borsch 2004). In recent years, several new species have been described or reestablished for the genus in Brazil (Bove 2008; Fleischmann and Rivadavia 2009; Souza and Bove 2011; Baleeiro et al. 2015, 2019; Gonella and Baleeiro 2018; Guedes et al. 2019; Baleeiro et al. in prep.), revealing the potential for the discovery of new species even in a genus that had been thoroughly revised taxonomically in the late 20^th^ century (Taylor 1989). The late botanist Peter Taylor meticulously studied *Utricularia* for over 40 years, culminating in his elaborate monograph that considered ca. 600 published names for 214 accepted species (see Fleischmann 2012b).

During a field trip to perform a floristic inventory of the Campos do Ariramba, an area of *campinarana* and savanna at the westernmost point of the state of Pará, several new records of Lentibulariaceae were made, including two collections of *Utricularia* that did not fit any of the currently recognized species. Here we describe these two new taxa and provide comments on their taxonomy, habitat, distribution, and their conservation status. We also provide a list of the species of the family registered in the area to contribute to the knowledge of the Amazonian grassland biodiversity, still so underestimated.

## Material and methods

An expedition to Campos do Ariramba region (Municipality of Óbidos) was carried out in the period between 5–10 June 2019. Specimens were collected and deposited in the herbarium MG with duplicates sent to SPF. Specimens of the herbaria ALCB, B, BHCB, BM, DIAM, ESA, ESAL, F, HUEFS, HUFSJ, HUFU, HURB, IAN, INPA, IPA, K, M, MG, MBM, MBML, MO, NY, OUPR, P, R, RB, SP, SPF, UB, UEC, UFRN, US, and VIES were also studied as part of the ongoing taxonomic study of the family for the Flora of Brazil 2020 project. The online databases Reflora Virtual Herbarium (REFLORA 2020), SpeciesLink (INCT 2018), and Global Biodiversity Information Facility (GBIF.org 2020) were also searched for further specimens of these taxa and for other specimens from Campos do Ariramba, Jaramacaru (including orthographic variants), Óbidos and Oriximiná. The descriptions were based on live material and dry specimens, which were analyzed using a stereomicroscope. Herbarium acronyms follow Thiers (2020 continuously updated).

For SEM photography, seeds from herbarium specimens were mounted on a carbon sticker-covered SEM stub, coated with platinum for 240 sec. under vacuum in a SCD 050 sputter coater (Bal-Tec, Germany), and imaged under SEM (Leo, Germany) at 25 mm working distance and 15.00 kV.

The distribution map was generated with the software QGIS (QGIS Development Team 2020) using layers available from IBGE (2020). The TerraBrasilis platform (Assis et al. 2019), which uses the Sistema de Detecção do Desmatamento em Tempo Real (DETER) of the Instituto Nacional de Pesquisas Espaciais (INPE), was used to acquire recent data on deforestation and fire records in the area. Coordinates were obtained in the field using GPS tracking. Given that the two species are known from a few locations each and lack population data, their conservation status were inferred based on criteria for the Area of Occupancy (AOO) of IUCN (2012), which was calculated by employing the IUCN standard 4 km^2^ cell in the GeoCAT Tool (Bachman et al. 2011).

Morphological terminology and description structure are adapted with modifications from Taylor (1989).

## Results

### Taxonomic treatment

#### Utricularia ariramba

Taxon classificationPlantaeLamialesLentibulariaceae

Gonella, Baleeiro & Andrino sp. nov.

0E6EF366-C8DA-5CCE-B159-54680FF6AC8F

urn:lsid:ipni.org:names:77213187-1

Figs 1 , 2 , 3

##### Type.

**Brazil. Pará**: Floresta Estadual de Trombetas, comunidade Jaramacaru, cachoeira do Rio Jaramacaru; 9 Jun. 2019; D.C. Zappi, C.O. Andrino, R.G. Barbosa-Silva & C. Maurity 4853 (holotype MG; isotype SPF).

**Figure 1.**
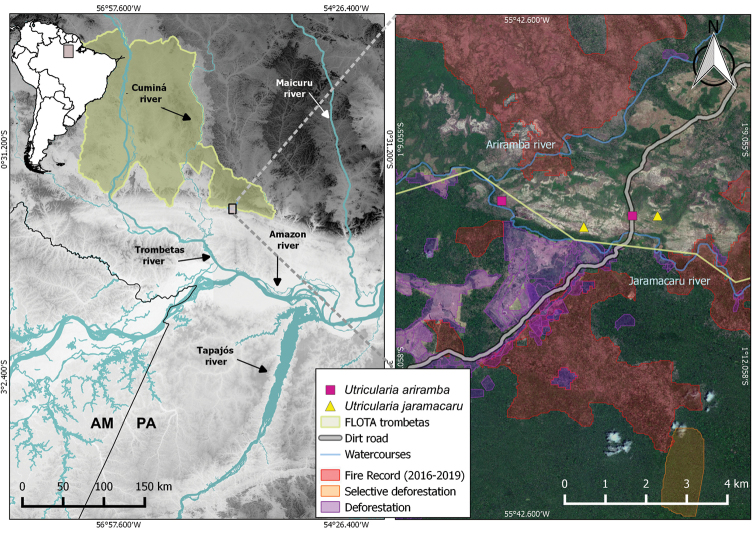
Distribution map of the new species of *Utricularia* in the Amazon. On the left map, the main rivers of the hydrographic basin of the region and which cross the FLOTA Trombetas (highlighted in green). The map to the right shows the records of *Utricularia ariramba* (squares) and *Utricularia jaramacaru* (triangle), which are near the FLOTA limits, as well the as threats to the area, including recent fires, full deforestation, and selective deforestation of timber species.

##### Diagnosis.

*Utricularia ariramba* belongs to U. sect Aranella (Barnhart) P.Taylor, being most similar to *U. costata* P.Taylor, but distinguished by its taller inflorescences 5.3–12.0 cm long (vs. 2–7 cm long), the less conspicuous nerves on the calyx lobes, the upper calyx lobe with acute apex (vs. obtuse and obscurely denticulate), spur swollen and dorsiventrally flattened in the apical 2/3 (vs. cylindrical with apex narrowing towards the tip), lower corolla lip trapezoid with margin entire or finely denticulate (vs. transversely oblong with margin entire or shallowly 3-lobed), and upper corolla lip narrowly ovate with acute apex (vs. ovate, with apex rounded or subacute).

##### Description.

Small-sized, probably annual, terrestrial. *Rhizoids* few, capillary, from the peduncle base, sometimes also from the basalmost scales, with short papillose branches, up to 0.8 mm long, 0.1–0.2 mm in diameter. *Stolons* few, capillary, terete, sparsely branched, a few cms long, c. 0.1 mm in diameter. *Leaves* few, on the stolons, petiolate, lamina narrowly linear, 1-nerved, up to 5 mm long, 0.1–0.2 mm wide. *Traps* numerous on the leaves and stolons, ovoid, stalked, 0.15–0.20 mm long, the mouth lateral with a single, conical, dorsal appendage and a longer, deeply bifid, ventral appendage. *Inflorescence* a bracteose raceme, erect, simple, solitary, 53–120 mm long; peduncle capillary, terete, glabrous, 0.2–0.3 mm in diameter, reddish-green. Scales, bracts, and bracteoles basifixed and single-nerved. *Scales* few to numerous, ovate-deltoid to ovate, with apex acute, 0.5–1.0 mm long. *Bracts* ovate-deltoid to lanceolate, with apex acute, 0.7–1.1 mm long. *Bracteoles* subulate, slightly shorter than the bract, with apex acute, 0.7–1.0 mm long. *Flowers* 1–6; pedicel ascending, filiform, terete, 0.5–2 mm long, c. 0.1 mm in diameter. *Calyx* lobes unequal, glabrous, with 9–13 very conspicuous, simple, parallel, raised nerves, green to greenish-red in color; upper lobe ovate with apex acute, convex, 2.0–2.5 × 1.2–1.5 mm; lower lobe ovate, convex, with apex bifid, 2.6–3.3 × 1.5–2.0 mm. *Corolla* white or lavender, with the lower lip with a yellow-orange mark on the top and violet nerves on the limb, 8–11 mm long, finely papillose; *upper lip* narrowly ovate 4.0–5.5 × 2–3 mm, apex acute, lateral margins retroflexed, basal sac with ciliated rim; *lower lip* limb trapezoid, margin entire or finely denticulate, straight or retroflexed, shallowly 3 lobed, 3–5 × 3–7 mm, palate with papillose rim; *spur* conical, swollen and dorsoventrally flattened in the apical 2/3, the apex truncate to sub-acute, projected to the front, longer than and +/- parallel to the lower lip, 5.0–6.1 × 2.2–3.1 mm, papillose and finely ciliated. *Filaments* curved, c. 0.8 mm long, the anther thecae sub-distinct, anther c. 0.70 × 0.25 mm. *Ovary* globose, 0.5 mm long; style short, c. 0.2 mm long; stigma bilabiate, lower lip semicircular, 0.3 mm wide, upper lip broadly deltoid. *Capsule* and *seeds* not seen.

##### Etymology.

The epithet “*ariramba*” is a name in apposition, referring to the Campos de Ariramba, where this new species was discovered. The word “*ariramba*” comes from the Tupi language “*uarirámba*” and refers to the birds of the Galbulidae family, which are commonly found in the area.

##### Phenology.

The species was collected in full bloom at the end of the rainy season, in May and June.

##### Distribution and habitat.

So far, only known from two subpopulations at the margins of the Jaramacaru River, in the Campos do Ariramba region. The area lies within the conservation unit of the Floresta Estadual de Trombetas (FLOTA Trombetas), in western Pará state, N Brazil. The species occurs on white sandy soils on a flat sandstone outcrop in *campinarana* (white sand vegetation).

##### Conservation status.

Vulnerable: VU D2. *Utricularia ariramba* is known from only three collections, one of which was made over 60 years ago and lacked georeferenced data. The recently collected specimens were found ca. 3 km distant from each other, near the border of the FLOTA Trombetas (Fig. 1). Areas under active deforestation were observed just outside the conservation unit (Fig. 1), and during the fieldwork activities in 2019, fire was observed in a waterfall 6 km upriver from the Jaramacaru community, suggesting environmental disturbance generated by cattle farming and other human activities. In fact, large areas were recently impacted by fires less than 1 km away from the subpopulations (Fig. 1; data from Assis et al. 2019). In addition, the site is sought by tourists from nearby municipalities because of the Jaramacaru waterfall, therefore leading to negative anthropogenic impact on the populations. Although the Campos do Ariramba are a botanically unexplored region with vast areas of habitats similar to those where the species was collected and the population size has not been ascertained, the available data suggests that the species is restricted to a few locations. An AOO of just 8 km^2^ was calculated for this species, and we observed threats that might impact its habitat quality and AOO in the short term and lead to a reduction in population size and area of occupancy. Therefore, we assign the species to the Vulnerable category based on criterion D2 of IUCN (2012).

##### Taxonomic notes.

*Utricularia ariramba* is placed in U. sect. Aranella based on its characteristic trap morphology (a single subulate dorsal appendage and a deeply bifid ventral appendage), and the presence of a clearly defined basal sac in the upper corolla lip.

*Utricularia ariramba* is the eleventh species of Utricularia sect. Aranella (Taylor 1989; Fleischmann and Rivadavia 2009), a section almost completely endemic to tropical South America, with a single species (*U. simulans* Pilger) extending to Central America, the Caribbean and tropical Africa (Taylor 1989).

*Utricularia ariramba* is most similar to *U. costata*, sharing similar bract and bracteole morphology, and the lavender (to white) corolla with darker violet venation in the lower lobe. It is distinguished by the relatively larger inflorescences 5.3–12.0 cm tall (vs. 2–7 cm tall), the less prominent nerves on the calyx lobes, the upper calyx lobe with acute apex (vs. obtuse and obscurely denticulate), the swollen spur in the apical 2/3 (vs. apex tapering towards the tip), the lower corolla lip trapezoid with margin entire or finely denticulate (vs. transversely oblong with margin entire or shallowly 3-lobed), and the upper corolla lip narrowly ovate with acute apex (vs. ovate, with apex rounded or subacute). For photos of *U. costata*, see Costa et al. (2016: 11, Fig. 3D, E) and Mota and Zappi (2018: 126, Fig. 3a–c).

*Utricularia costata* occurs in Venezuela and Brazil, where it is recorded from the states of Roraima, Pará, Mato Grosso, Goiás, Bahia, Sergipe and Alagoas (Taylor 1989, 1999; Fleischmann and Rivadavia 2009; Carregosa and Costa 2014; Costa et al. 2016; Guedes et al. 2018; Flora do Brasil 2020 under construction). In Pará, the species is recorded from the southeast region of the state, in the Serra dos Carajás (Mota and Zappi 2018).

**Figure 2.**
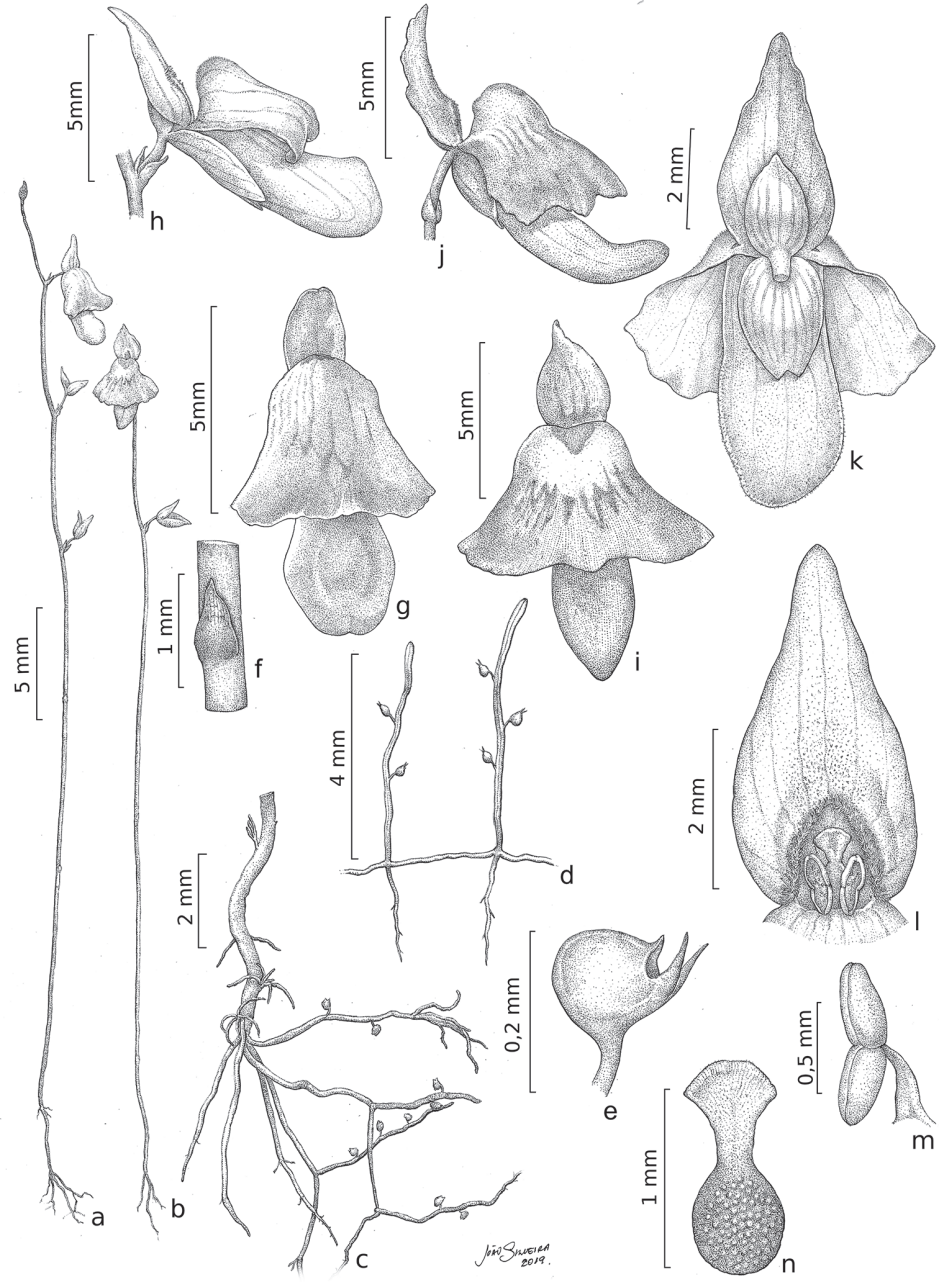
*Utricularia ariramba***a, b** habit, in flower **c** base of plant with stolons, rhizoids, and peduncle base **d** part of stolon, with rhizoids, and leaves with traps **e** utricle, side view **f** scale **g, h** flower of the white morphotype in anterior (**g**) and side (**h**) view **i, j** flower of the lavender morphotype in anterior (**i**) and side (**j**) view **k** flower of the white morphotype in posterior view **l** upper lip of the corolla, androecium, and gynoecium **m** stamen **n** pistil **a, c–h, k–n** based on the holotype; **b, i, j** based on *C.O. Andrino 560*. Illustrations by João Silveira.

**Figure 3.**
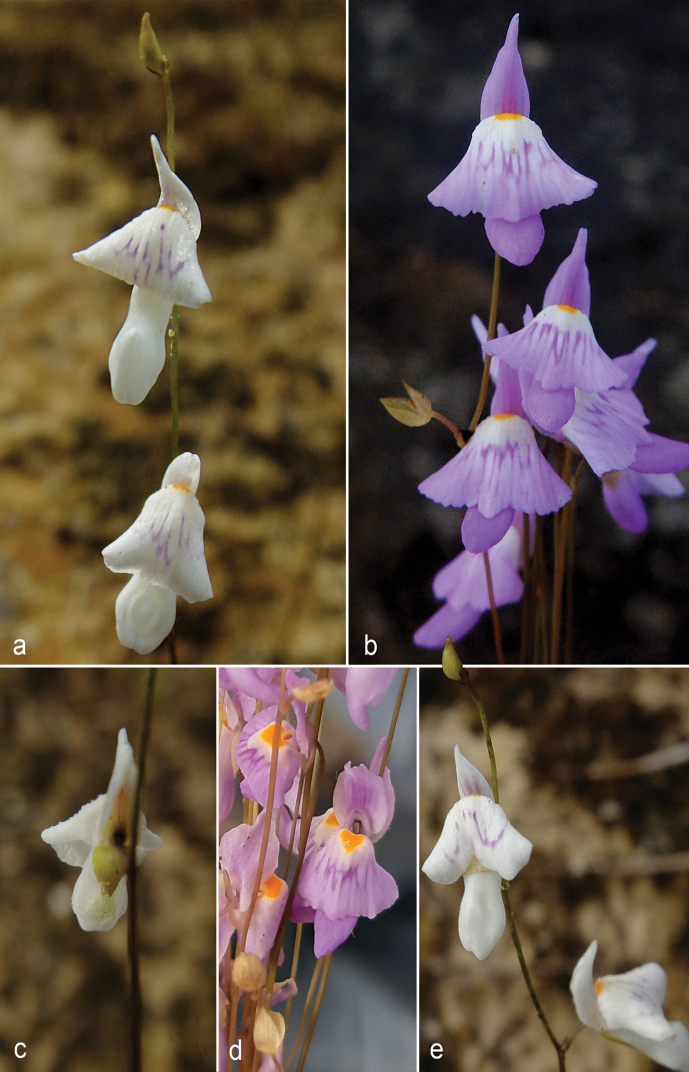
*Utricularia ariramba***a** inflorescence apex with flowers and bud of the white corolla morphotype, showing the reflexed apex of lower corolla lip **b** flowers of the lavender corolla morphotype, with a calyx of a developing fruit to the left **c** flower of the white corolla morphotype in posterior view, showing the calyx lobes and the concavity in the ventral portion of the spur **d** flowers of the lavender corolla morphotype **e** inflorescence apex of the white corolla morphotype showing variation in spur morphology.

Variation in corolla color and spur morphology was observed in the studied specimens of *U. ariramba*, where it varies from white to lavender, and the different corolla colors are associated with different spur shapes: both variants show a dorsoventrally flattened spur in the apical 2/3, but the white variant (represented by the type specimen) shows a higher degree of flattening, a ventral concavity (Fig. 2c), and a truncate apex (Fig. 3a), while the lavender variant (represented by the paratypes) has a spur only slightly flattened, without the concavity, and with an acute apex (Figs 2i, 3b, d). Furthermore, in the white morphotype, the apex of the lower corolla lip is reflexed (Figs 2h, 3a). Despite these differences in corolla color and shape, both morphotypes are considered conspecific as the specimens share similar morphology in all other characters. Variation in corolla color and shape is common in other species of U. sect. Aranella, as exemplified by intra-populational variation observed in *U. laciniata* A.St.-Hil. & Girard and *U. purpureocaerulea* A.St.-Hil. & Girard in several areas across their range (PMG pers. obs.), as well as in lavender- and white-flowered color morphs of *U. blanchetii* A.DC. in the Chapada Diamantina (Taylor 1989; Rivadavia 2000).

Seeds were not available for study, however, Taylor (1989: 237) notes that seed morphology is rather uniform in U. sect. Aranella, hence not having great taxonomic significance (compared to other sections of the genus, where some species might be identified by a single seed grain alone; Taylor 1964, 1989).

##### Additional specimens examined

**(paratypes).** Brazil • Pará; [Óbidos]; Campo do Jamaracarú [*sic*], perto do barracão, região do Ariramba; 26 May 1957; G.A. Black, W. Egler, P. Cavalcante & A. Silva 57-19633 (IAN 95750) • Óbidos; FLOTA Trombetas, comunidade Jaramacaru; 10 Jun. 2019; C.O. Andrino 560 (MG, SPF).

### Key to Utricularia sect. Aranella

Adapted from Taylor (1989: 238) and Fleischmann and Rivadavia (2009: 158) with the addition of *U. ariramba* as follows:

**Table:** 

7	Lowermost scales deeply fimbriate	***U. laciniata***
7’	Lowermost scales entire	**8**
8	Upper corolla lip narrowly ovate with acute apex; spur of the corolla swollen and dorsoventrally flattened in the apical 2/3	***U. ariramba***
8’	Upper corolla lip transversally elliptic, quadrate or ovate, with apex rounded to crenate; spur of the corolla tapering towards the tip and never dorsoventrally flattened	**9**
9	Spur of corolla with apex obtuse, 2–3 times as long as the lower lip; upper lip scarcely longer than wide; calyx very strongly nerved, the lower lobe longer	***U. costata***
9’	Spur of corolla with apex acute or shortly bifid, shorter or scarcely longer than the lower lip; calyx not very strongly nerved, the lobes ± equal or the upper lobe longer at anthesis	**10**
10	Upper lip of corolla not wider than calyx, quadrate to elliptic, scarcely longer than wide, constricted below the middle; spur curved upwards; calyx upper lobe with apex acute, enclosing the capsule in fruit; seed truncate-obovoid	***U. rostrata***
10’	Upper lip of corolla much wider than calyx, transversely elliptic, much wider than long, not constricted; spur straight; calyx upper lobe with apex 3-dentate, not enclosing the capsule in fruit; seed ovoid	***U. purpureocaerulea***

#### Utricularia jaramacaru

Taxon classificationPlantaeLamialesLentibulariaceae

Gonella, Baleeiro & Andrino sp. nov.

B77BE6FD-59F8-5D16-83D9-E3D29B6B4FFB

urn:lsid:ipni.org:names:77213188-1

Figs 1 , 4 , 5 , 6

##### Type.

**Brazil. Pará**: Óbidos; Floresta Estadual de Trombetas, Ariramba, Rio Jaramacaru; 10 Jun. 2019; C.O. Andrino, R.G. Barbosa-Silva, D.C. Zappi & C. Maurity 559 (holotype MG; isotypes M, SPF).

**Figure 4.**
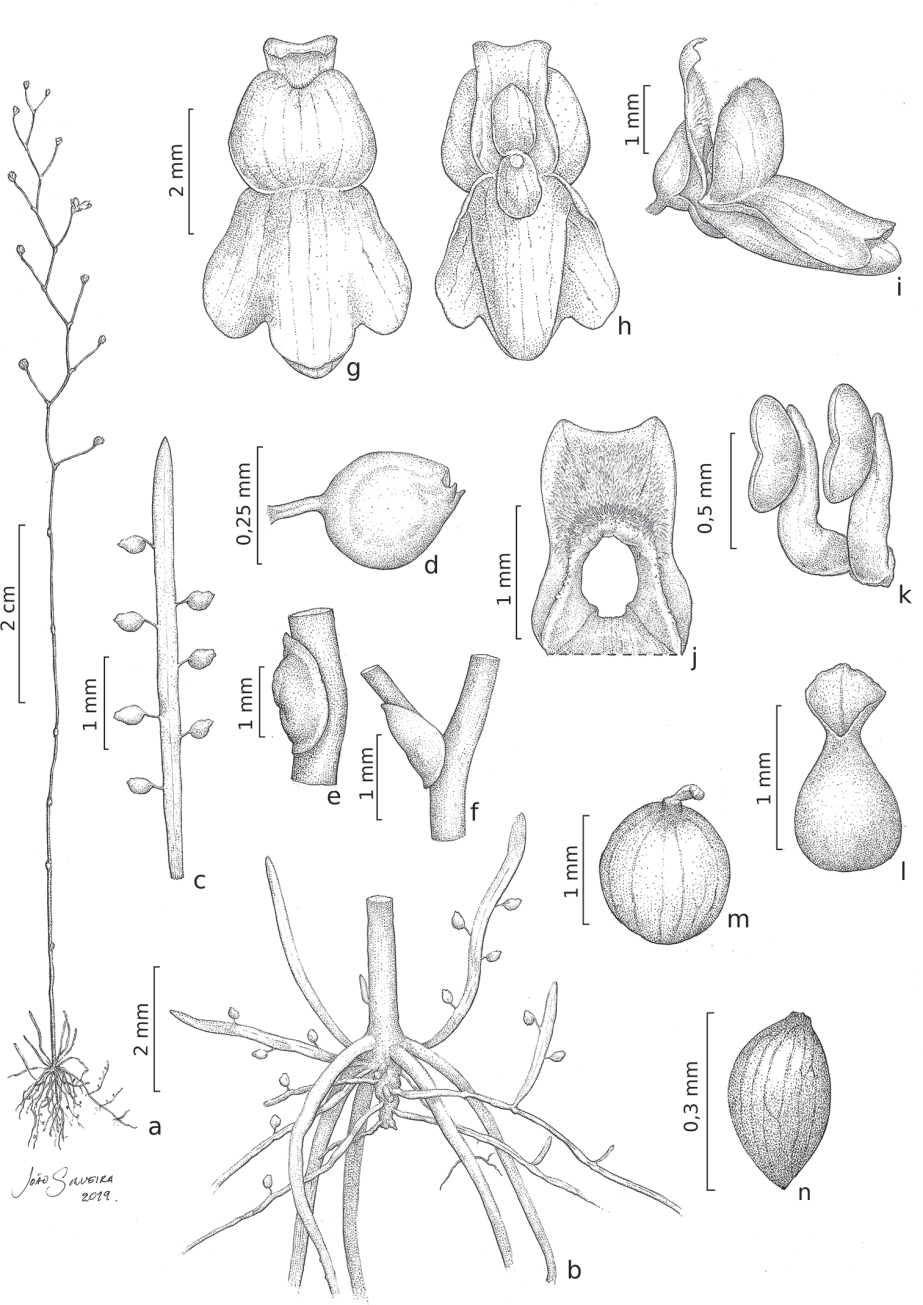
*Utricularia jaramacaru***a** habit, in flower **b** base of plant with stolons with traps, rhizoids, leaves with traps, and peduncle base **c** leave with traps **d** utricle, side view **e** scale **f** base of the pedicel, and bract **g** flower, in anterior **h** flower, in posterior view **i** flower, in lateral view **j** upper lip of the corolla **k** stamens **l** pistil **m** fruit **n** seed. All based on the holotype. Illustrations by João Silveira.

##### Diagnosis.

*Utricularia jaramacaru* belongs to U. sect. Setiscapella (Barnhart) P.Taylor but is distinct from all other members of this section by the traps with reduced, denticulate appendages (vs. subulate, branched), white corolla (vs. yellow or lilac), the upper corolla lip with bilobate apex (vs. obtuse, rounded, truncate or retuse), and the lower corolla lip narrowly rhombic (vs. cuneate, trullate, rhombic to very broadly rhombic in outline).

##### Description.

Small-sized, probably annual, terrestrial. *Rhizoids* 2–4, from the base of peduncle, terete, with short papillose branches, up to 1 cm long, c. 0.25 mm in diameter. *Stolons* numerous, capillary, sparsely branched, up to 1 cm long (in the available material), up to 0.1 mm in diameter. *Leaves* numerous, at the base of the peduncle and on the stolons, lamina narrowly linear, simple, the base narrowing gradually into a short petiole, apex obtuse to acute, green to reddish, 1-nerved, 2–6 × 0.2–0.5 mm. *Traps* numerous on the stolons and leaves, ovate, stalked, 0.1–0.2 mm long, the mouth lateral with two dorsal and very short denticulate, simple appendages. *Inflorescence* a bracteose raceme, erect, solitary, 60–130 mm tall. *Peduncle* capillary, terete, simple or eventually laterally simple-branched, glabrous, 0.2–0.3 mm in diameter, wine red. *Scales* numerous, peltate, ovate to narrowly ovate, inferior apex rounded to obtuse, superior apex acute, 0.5–0.9 mm long, similar to the bracts. *Bracts* ovate, basisolute, peltate, 0.5–0.7 × 0.4–0.5 mm, amplexicaul, the inferior apex rounded, the superior apex rounded to obtuse. *Bracteoles* absent. *Flowers* 4–13, the rhachis elongate, flexuous, without sterile bracts; pedicels ascending, capillary, terete, 3–9 mm long (longer towards the base of the inflorescence), pedicels with a mucilage droplet at their base in living specimens. *Calyx* lobes unequal, glabrous, nerves inconspicuous, simple, not extending to the margin; upper lobe ovate, with apex obtuse, convex, 0.9–1.1 mm long in flower, up to 1.3 mm in fruit; lower lobe obovate, with apex emarginate to rounded, convex, equal in length with the upper lobe in flower, slightly longer in fruit, up to 1.7 mm in fruit. *Corolla* 5 mm long, lower lip white with a pale yellow mark on the gibbose palate, spur pale yellow, upper lip pale yellow with reddish marks; upper lip oblong with apex bilobed, the basal sac with an eglandular pubescent marginal rim, the pubescence spreading towards the apex, c. 1.5 mm long; lower lip limb narrowly rhombic in outline, the base with a very prominent bilobed swelling, the apex 3-lobed, 0.3–4.5 mm; palate pubescent; spur cylindrical, apex rounded, equal to or slightly longer or shorter than the lower lip, 0.35–0.40 mm long. *Filaments* curved, 0.8–1.0 mm long, the anther thecae sub-distinct, anther 0.4–0.5 mm long. *Ovary* globose, 0.8–0.9 mm long; style very short; stigma lower lip nearly circular, upper lip obsolete. *Capsule* globose, c. 1.2 mm in diam., shorter than the calyx lobes, dehiscing by an elliptic ventral pore. *Seeds* obovoid to angulate-ellipsoid, 0.20–0.25 mm long, 0.13–0.20 mm wide, testa cells c. 0.01 mm wide, elongate, anticlinal boundaries deeply sunken and more or less straight, periclinal walls convex, smooth.

##### Etymology.

The epithet “*jaramacaru*” is a noun in apposition (hence it is invariant), referring to the Jaramacarú river, where the new species was discovered. “Jaramacarú” comes from the Tupi language “*iamandakarú*”, referring to species of the genus *Cereus* Mill. (Cactaceae). However, no cactus of this genus was located during the field trip undertaken by COA, RGBS, and DCZ in 2019.

##### Phenology.

*Utricularia jaramacaru* was collected with flowers in April, May, and June.

##### Distribution and habitat.

So far, only known from two very close localities near the Jaramacaru waterfall, in the Campos do Ariramba, part of the FLOTA Trombetas, western Pará, N Brazil. The species occurs on white sandy soils with outcrops of sandstone, in *campinarana* vegetation.

##### Conservation status.

Vulnerable: VU D2. Similarly to that described for *U. ariramba*, *U. jaramacaru* is known from only two localities (AOO=8 km^2^) near the limits of FLOTA Trombetas and the threats the populations are subject to are fully explained in the above species. Therefore, based on available data, *U. jaramacaru* is to be assigned to the category of Vulnerable based on criterion D2 of IUCN (2012).

##### Taxonomic notes.

**Figure 5.**
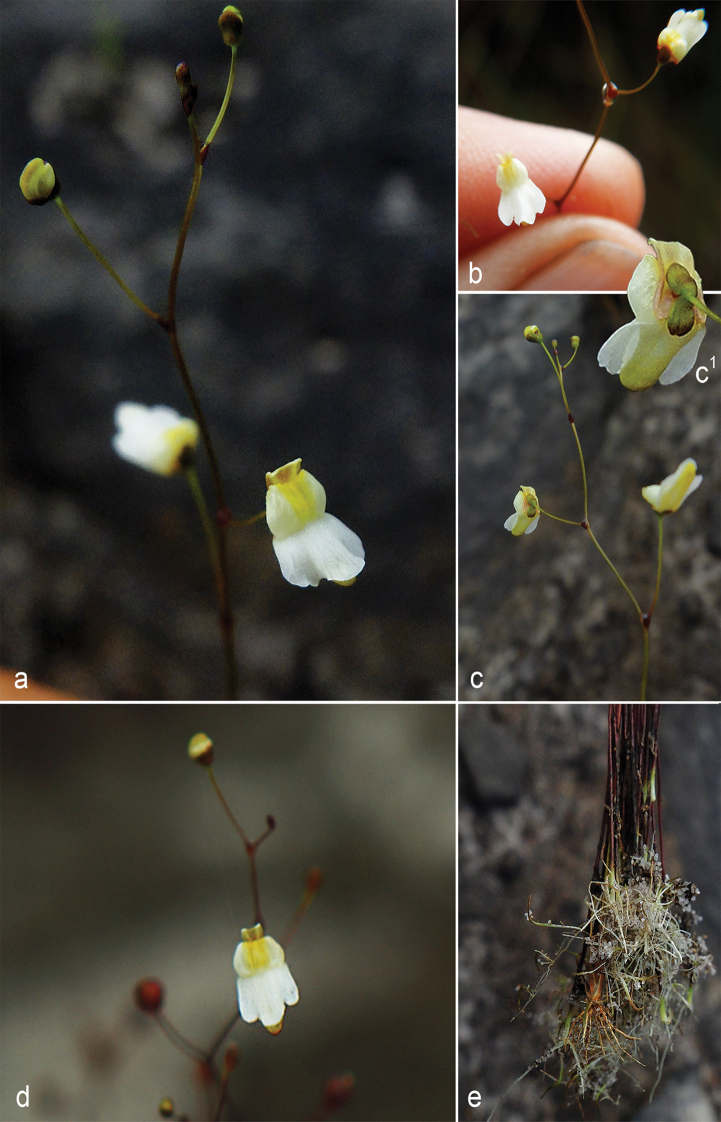
*Utricularia jaramacaru***a** inflorescence apex with open flowers and bud **b** detail of inflorescence apex showing mucilage droplet in the axil of a pedicel **c** inflorescence apex with a flower in posterior view, highlighting the calyx (**c1**) **d** flower in anterior view; **e**, peduncle bases with stolons and leaves.

**Figure 6.**
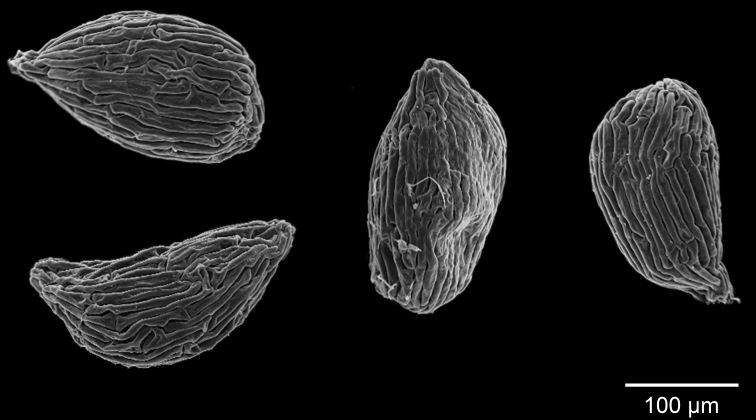
SEM microphotographs of seeds of *Utricularia jaramacaru* at 420× magnification, in dorsal, lateral and oblique view (from the holotype).

The basisolute, peltate scales and bracts, and the calyx and seed morphology (Figs 4–6) undoubtedly place this species in U. sect. Setiscapella, representing the tenth species of the section (following the species circumscriptions of Taylor 1989). However, based on morphology alone, it is not possible to assign the closest affinity of *U. jaramacaru*, as it bears several apomorphic characteristics, most remarkably regarding its trap and corolla morphology.

Up to now, U. sect. Setiscapella was composed of nine species (Taylor 1989), of which eight have yellow corollas (regarding the phylogenetic switch from lilac to yellow corolla color in *Utricularia* and *Genlisea*, see Fleischmann et al. 2010). One exception in terms of color is *U. physoceras* P.Taylor, also endemic to the state of Pará, but with larger (7–10 mm long vs. 5 mm) pink to lilac corolla. The whitish corolla of *U. jaramacaru* is, therefore, a second exception among the species of the section. *Utricularia physoceras* also shares the short spur with rounded apex with *U. jaramacaru* and similar seed morphology. *Utricularia physoceras* occurs in the *cangas* (ferruginous *campo rupestre*) of the Serra dos Carajás (Taylor 1989; Mota and Zappi 2018; Giulietti et al. 2019), distant ca. 815 km to the southeast from the area where *U. jaramacaru* was collected. For photos of *U. physoceras*, see Mota and Zappi (2018: 129, Fig. 4a–e) and Giulietti et al. (2019: 369, fig. 7 bottom three images).

Traps of *U. jaramacaru* are unlike any other species of U. sect. Setiscapella in that the appendages are reduced to two denticulate structures (Fig. 4d). All other species of the section bear subulate or filiform appendages near the trap door that are sparsely to copiously branched. Reduced appendages are found in different sections of *Utricularia*, which suggests it is a homoplastic character in the genus. Taylor (1989) enumerates a few species with reduced trap appendages, such as *U. cornuta* Michx. and *U. juncea* Vahl (both of U. sect. Stomoisia (Raf.) Komiya), *U. nana* A.St.-Hil. & Girard (U. sect. Benjaminia P.Taylor), *U. guyanensis* A.DC. (U. sect. Stylotheca A.DC.) and *U. viscosa* Spruce ex Oliv. (U. sect. Sprucea P.Taylor), all presenting only a small prolongation of the body of the trap on the dorsal side of the door.

The presence of a droplet of mucilage at the insertion of the pedicel in the peduncle (Fig. 5b) is shared with *U. flaccida*, *U. nigrescens* Sylvén and *U. pusilla* Vahl (P.M. Gonella and A. Fleischmann, pers. obs.), and its function remains unclear.

##### Additional specimens examined

**(paratypes)**. Brazil • Pará; [Óbidos]; Rio Jaramacaru, entre o acampamento e a cachoeira; 26 May 1957; G.A. Black, W. Egler, P. Cavalcante & A. Silva 57-19500 (IAN 95620) • *ibid*.; trilha após a ponte do rio Jamaracaru [*sic*], em direção à cachoeira; 01°10'12.99"S, 055°41'50.69"W; 80 m; 27 Apr. 2018; J.A. Siqueira Filho 4112 (HVASF 23703).

### Key to Utricularia sect. Setiscapella

Adapted from Taylor (1989: 523) with the addition of *U. jaramacaru* as follows:

**Table:** 

1	Corolla pink, lilac or white	**2**
1'	Corolla yellow	**3**
2	Trap with filiform and sparsely branched appendages; corolla pink or lilac, 7–10 mm long, upper lip apex rounded or truncate	***U. physoceras***
2'	Trap with denticulate and simple appendages; corolla white, c. 5 mm long, upper lip apex bilobed	***U. jaramacaru***

### List of other Lentibulariaceae species recorded in the Campos do Ariramba

Below, we present the preliminary list of the Lentibulariaceae from the Campos do Ariramba region followed by the specimen(s) examined, comprising 12 species (including the new species here described). This number is certainly an underestimation of the family’s diversity in the area, as we still believe the site remains under-sampled. Further fieldwork covering a broader area and different months are deemed necessary to present a more comprehensive list in the future.

*Genlisea oxycentron* P.Taylor

Brazil. Pará: Óbidos; FLOTA Trombetas, Comunidade Jaramacaru, Rio Jaramacaru; 10 Jun. 2019; C.O. Andrino 558 (MG).

*Utricularia adpressa* Salzm. ex A.St.-Hil. & Girard

Brazil. Pará: Óbidos; FLOTA Trombetas, Estrada para Tabuleta, Campos do Ariramba; 11 Jun. 2019, R.G. Barbosa-Silva 1167 (MG) • *ibid*; comunidade Jaramacaru, Estrada para a Cachoeira do Rio Jaramacaru; 12 Jun. 2019; C.O. Andrino 572 (MG) • *ibid*; Campos Gerais (Ariramba), a cerca de 5 km da ponte do Jaramacarú em linha reta; 9 Jun. 2019; J.F. Maciel-Silva 400 (MG).

*Utricularia amethystina* Salzm. ex A.St.-Hil. & Girard

Brazil. Pará: Óbidos; trilha após a ponte do rio Jamaracaru [*sic*] em direção à cachoeira; 01°10'05.49"S, 055°42'35.39"W; 76 m; 27 Apr. 2018; J.A. Siqueira Filho 4115 (HVASF 23706); • *ibid*; FLOTA Trombetas, comunidade Jaramacaru, Cachoeira do Rio Jaramacaru; 9 Jun. 2019; D.C. Zappi 4846 (MG) • *ibid*; comunidade Jaramacaru, Rio Jaramacaru; 10 Jun. 2019; C.O. Andrino 557 (MG) • *ibid*; Estrada para Tabuleta, Campos do Ariramba; 11 Jun. 2019; R.G. Barbosa-Silva 1149 (MG) • *ibid*; Campos Gerais (Ariramba), à cerca de 5 km da ponte do Jaramacarú em linha reta; 9 Jun. 2019; J.F. Maciel-Silva 402 (MG) • *ibid*; Campos Gerais (Ariramba), campina a 300 m a nordeste da cachoeira do Jaramacarú; 7 Jun. 2019; C.S. Nunes 486 (MG).

*Utricularia hispida* Lam.

Brazil. Pará: Região do Ariramba, Igarapé Quebra-dente; 30 May 1957; G.A. Black et al. 19802 (IAN 96255).

*Utricularia hydrocarpa* Vahl

Brazil. Pará: Óbidos; FLOTA Trombetas, comunidade Jaramacaru, Cachoeira do Rio Jaramacaru; 9 Jun. 2019; D.C. Zappi 4836 (MG) • *ibid*; Campo do Jamaracaru [*sic*], perto do barracão, região do Ariramba; 26 Jun. 1957; G.A. Black et al. 19620 (IAN 95738).

*Utricularia longeciliata* A.DC.

Brazil. Pará: Oriximiná; Campos do Ariramba, campinas inundáveis da margem do Rio Jaramacarú, afloramentos areníticos; 70 m a.s.l.; 08 Jun. 1980; G. Martinelli et al. 6880 (RB 203406) • *ibid*, G. Martinelli et al. 6897 (RB).

*Utricularia neottioides* A.St.-Hil. & Girard

Brazil. Pará: Óbidos; FLOTA Trombetas, comunidade Jaramacaru, Estrada para Tabuleta, Campos do Ariramba, Igarapé do Mutum; 11 Jun. 2019; R.G. Barbosa-Silva 1134 (MG).

*Utricularia pusilla* Vahl

Brazil. Pará: Óbidos; FLOTA Trombetas, comunidade Jaramacaru, Beira da Cachoeira do Rio Jaramacaru; 12 Jun. 2019; C.O. Andrino 584 (MG);

*Utricularia simulans* Pilg.

Brazil. Pará: Óbidos; FLOTA Trombetas, Estrada para Tabuleta, Campos do Ariramba; 7 Jun. 2019; D.C. Zappi 4799 (MG).

*Utricularia subulata* L.

Brazil. Pará: Óbidos; perimetral norte, Rio Jaramacaru, área alagada entre os afloramentos rochosos próximo à casa de Juarez; 01°10'16.89"S, 055°41'16.39"W; 80 m a.s.l.; 27 Apr. 2018; J.A. Siqueira Filho 4107 (HVASF 23698) • *ibid*; FLOTA Trombetas, comunidade Jaramacaru, Cachoeira do Rio Jaramacaru; 9 Jun. 2019; D.C. Zappi 4851 (MG) • *ibid*; Rio Jaramacaru; 10 Jun. 2019; C.O. Andrino 561 (MG) • *ibid*; Floresta Estadual do Trombetas, Campos Gerais (Ariramba), cerca de 1 km a sul da Vila do Jaramacarú; 11 Jun. 2019; M. Pastore 947 (MG) • Oriximiná; Campos de Ariramba, Campinas inundáveis da margem do afloramento arenítico; 8 Jun. 1980; G.M. Martinelli 6878 (INPA, MG).

## Discussion and concluding remarks

Both species described here were first collected over 60 years ago, and the specimens remained undetermined until the preparation of this work. This is not an isolated event since more than half of the new species described in recent years were published decades after being first collected and deposited in herbarium collections (Bebber et al. 2010). In the case of these new *Utricularia* species, a few factors can be listed to explain the lag between first collection and description, which are common in other plant groups. First, Amazonian herbaria are quite distant from most botanical research centers, receiving fewer resources, both human and financial, therefore being less visited by specialists (Hopkins 2019; de Gasper et al. 2020). This is also reflected by the still low number of imaged specimens in the collections housed in these herbaria, hindering the remote access by specialists and leaving a considerable number of these specimens ‘undetermined’. This means that the average number of species not yet described deposited in these herbaria tends to be higher, accentuating the urgent debate on taxonomy in describing diversity in the current biodiversity crisis (Dubois 2003; Giam et al. 2011; Tedesco et al. 2014). Also, it is worth highlighting that, in this case, the identification of the historical herbarium specimens as new species could only be confirmed after new collections were made, therefore reinforcing both the importance of funding for fieldwork in remote areas and the relevance of such herbaria collections as sources for identification of the still undescribed diversity (for further examples of similar cases, see: Ferreira et al. 2016; Barbosa-Silva et al. 2018; Farroñay et al. 2018).

Similarly to several areas of open vegetation in the Amazon and the Amazon rainforest itself, the vegetation of the Campos do Ariramba is poorly understood, and, until recently, few botanical expeditions were carried out to the area. The most significant botanical contributions to the area were conducted by Adolpho Ducke in 1905 and 1906, resulting in the description of several new species for the region, such as *Dyckia duckei* L.B.Sm. (Smith 1958), *Ouratea duckei* Huber (Huber 1913), and *Caraipa myrcioides* Ducke (Ducke 1922). Later, another expedition conducted by Walter A. Egler and George Alexander Black resulted in the first and only preliminary floristic list published for the area (Egler 1960). Egler (1960) makes it clear that the list is unfinished because Black died tragically after the expedition, and his material was not found, disappearing together with his valuable observations and field records that possibly resulted in the aforementioned gaps in Egler’s study, which was dedicated posthumously to Black. An example of these gaps are the Lentibulariaceae, which are cited in the text as important elements of the wetlands but not listed in the work (Egler 1960). This further justifies the presentation of a full list for the family herewith.

The access to the Campos do Ariramba, currently only possible by dirt road, is the result of a failed attempt to connect the site to the savannas in the northern limit of Pará state (called by Ducke and Egler as Campos Gerais; currently the Tumucumaque Indigenous Park), intending to create areas for livestock (Ducke 1910; Egler 1960). The construction of this road has impacted the area through deforestation and modification into cattle pastures adjacent to the road to the FLONA (Fig. 1). This is not an isolated occurrence in the municipalities of Óbidos and Oriximiná, as roads intensify deforestation in the Amazon, while protected areas mitigate this impact (Pfaff et al. 2007; Barber et al. 2014). This scenario, coupled with the current environmental policy that is incapable or unwilling to preserve the Amazon Rainforest biome is increasing deforestation and accelerating climate change (Rajão et al. 2020), representing a poor prospect especially for range-restricted, inconspicuous species that might be extinct even before being collected and identified.

## Supplementary Material

XML Treatment for Utricularia ariramba

XML Treatment for Utricularia jaramacaru
